# Ground-glass opacity score predicts the prognosis of anti-MDA5 positive dermatomyositis: a single-centre cohort study

**DOI:** 10.1186/s13023-023-02827-x

**Published:** 2023-07-21

**Authors:** Lijun Liu, Yinli Zhang, Cong Wang, Wenjuan Guan, Xin Zhang, Lei Zhang, Yujie He, Wenlu Hu, Shengyun Liu, Tianfang Li

**Affiliations:** 1 Department of Rheumatology, The First Affiliated Hospital of Zhengzhou University, E. Jianshe Rd. 1, Zhengzhou, Henan 450000 China; 2 Department of Gynecology, The First Affiliated Hospital of Zhengzhou University, Zhengzhou, China

**Keywords:** Ground-glass opacity score, Anti-melanoma differentiation-related gene 5, Dermatomyositis

## Abstract

**Objective:**

Dermatomyositis (DM) positive with anti-melanoma differentiation-associated gene 5 (anti-MDA5-DM) is a systemic autoimmune disease with high mortality. This study aimed to explore the risk factors of death in anti-MDA5-DM and validate a prediction model for all-cause mortality in anti-MDA5-DM.

**Method:**

We conducted a retrospective study using a single-centre cohort of patients with newly onset anti-MDA5-DM from June 1, 2018 to August 31, 2021. Patients were divided into four groups according to baseline ground-glass opacity (GGO) score: Group A, GGO ≤ 1; Group B, 1 < GGO ≤ 2; Group C, 2 < GGO ≤ 3; Group D, GGO > 3. The primary outcome was death during the follow-up. Secondary outcomes included death within 3, 6, 12 months, severe infection, and remission during the first 12 months.

**Results:**

A total of 200 patients were included in the study. Based on multivariable Cox regression, the prognostic factors at baseline were identified as CRP > 5 mg/L, serum ferritin (SF) > 600ng/ml, positive anti-Ro52 antibody, prophylactic use of compound sulfamethoxazole (SMZ Co), four-category GGO score: GGO ≤ 1, 1 < GGO ≤ 2, 2 < GGO ≤ 3, GGO > 3. The final mortality of four groups was 16.4, 22.2, 48.5, 92.0%, respectively. Compared with Group A, the Hazards Ratio (HR) of Group B was 1.408, (p = 0.408), HR of Group C was 3.433 (p = 0.005), HR of Group D was 4.376 (p = 0.001).

**Conclusions:**

GGO score is a reliable predictor for risk stratification in anti-MDA5-DM and may provide guidance for individualized managements of patients.

## Rheumatology key messages

• GGO score is a simple and reliable predictor for risk stratification in anti-MDA5-DM.

• Baseline CRP > 5 mg/L, serum ferritin > 600 ng/ml and positive anti-Ro52 antibody are risk factors for death in anti-MDA5-DM patients, and prophylactic use of compound sulfamethoxazole is protective.

## Introduction

Anti-MDA5-DM is a systemic autoimmune disease with high mortality. Interstitial lung disease (ILD) is a common complication and a leading cause of death [1]. Anti-MDA5 antibody was a risk factor for rapidly progressive ILD (RPILD) [2, 3]. The mortality of anti-MDA5-DM is significantly increased when RPILD occurs [4–8].

Great endeavors have been made in recent years to stratify the prognosis of anti-MDA5 patients. Although no consensus has been reached, we recognize that some clinical, laboratory and imaging features are associated with prognosis. First, advanced age, ulcerative rash, and RPILD may be risk factors for mortality [9, 10]. Second, in terms of pulmonary function test, the FVC value is helpful for ILD assessment and risk stratification [11]. Among the serological indicators, higher titers of anti-MDA5 antibody, anti-Ro52 antibody positivity, and higher levels of CRP, lactate dehydrogenase (LDH), serum ferritin (SF) and KL-6 have been reported to be associated with mortality [7, 10, 12–15]. With regards to image-based parameters, high-resolution computed tomography (HRCT) scores have been implicated to be associated with the prognosis [7, 15, 16]. No consensus is currently available for the optimal treatment of anti-MDA5-DM. The most frequently used drugs included glucocorticoids (GC), calcineurin inhibitors and cyclophosphamide (CYC) [17, 18]. Studies have shown that initial intensive combination of immunosuppressants can improve prognosis [7, 12, 19]. In fact, the triple combination of sufficient doses of GC, CYC and high-dose tacrolimus are widely used in clinical practice [19]. Other studies indicate that the combination of tofacitinib may significantly benefit the patients [20–22].

The newly onset anti-MDA5-DM patients admitted to our center from June 1, 2018 to August 31, 2021 were followed-up for a median of 15 months in order to further explore the prognosis-related risk factors of anti-MDA5-DM and to provide a basis for risk stratification of such patients.

## Methods

### Patients

The study included patients with newly onset anti-MDA5-DM admitted to the First Affiliated Hospital of Zhengzhou University from June 1, 2018 to August 31, 2021. Patients with juvenile onset (n = 1), overlapping diseases (n = 6), poor compliance (n = 2) and coexisting malignancies (n = 5) were excluded. Treatment with low-dose glucocorticoids for less than one month was allowed. The clinical data were obtained from the medical system and followed up by phone. The diagnosis of DM was established based on Bohan and Peter criteria [23]. This study was approved by the Ethics Committee of the First Affiliated Hospital of Zhengzhou University (2022-KY-0427).

### Data collection

Data collection included demographic and clinical features at baseline, as well as treatment, survival and infections. Anti-MDA5 antibody was determined by the same laboratory and team using ELISA kits (MBL, Japan). Anti-Ro52 antibody was determined using lining immunofluorescence (Euroimmun, Germany). Baseline HRCT assessment was based on GGO score using the method proposed by Kazerooni, et al. [24]. The GGO score represented as the average extent of GGO (0: none, 1: < 5%, 2: 5 to < 25%, 3: 25 to < 50%, 4: 50 to < 75%, 5 :75% <) of five lobes. Patients were divided into four groups according to baseline GGO: Group A, GGO ≤ 1; Group B, 1 < GGO ≤ 2; Group C, 2 < GGO ≤ 3; Group D, GGO > 3. RPILD was defined as acute progressive dyspnea and hypoxemia within 4 weeks from the onset of respiratory symptoms, accompanied by the aggravation of ILD on HRCT [25].

### Treatment regimens

Pulse GC was defined as initial prednisone 1–2 mg/kg/day. Pulse intravenous immunoglobulin (IVIG) was defined as a total of 2 g/kg given in 3–5 days. The doses of calcineurin inhibitors (CNIs) were tacrolimus 3–4 mg/d or cyclosporine A 3-5 mg/kg/d. The doses of CYC were intravenous CYC: 0.4 g weekly or 0.6 g every other week, and oral CYC: 0.1 g/day. Tofacitinib was given as 10 mg/day. Prophylactic use of SMZ Co (each tablet contains 400 mg of sulfamethoxazole and 80 mg of trimethoprim) was defined as 2 tablets daily, 1 tablet daily, 1 tablet every other day, or 2 tablets twice a week. Pulse GC was administered to all patients immediately after diagnosis. Other agents include mycophenolate mofetil, rituximab and tocilizumab. According to the application of immunosuppressants, the treatment regimens were divided into 5 categories: GC + CNIs + CYC; GC + CNIs + tofacitinib; GC + CYC; GC + CNIs; other regimens.

### Outcomes

The primary outcome was designated as the death during the follow-up. Other outcomes were as follows: death within 3, 6, and 12 months, remission and serious infections within 12 months. Remission was defined as relief of clinical symptoms (not limited to respiratory symptoms), reduction or stabilization of GGO score on HRCT, and glucocorticoid dose of no more than prednisone 7.5 mg/day or its equivalents.

### Statistical analysis

Categorical variables were described as percentages. Continuous variables were tested for K-S normality. Variables with normal distribution were presented by mean ± standard deviation, and those with skewed distribution were described by median and quartiles. For continuous variables with missing values less than 20%, missing values were filled up with the expectation maximization method. Except for the GGO scores, other continuous variables were converted to dichotomous variables, and cutoff values were set according to the ROC analysis or medians, as appropriate. Patients’ baseline features, treatment and prognosis were compared among the four-category-GGO groups. Categorical variables were assessed by the Chi-squared test or Fisher’s exact test. Variables with normal distribution were assessed by univariate ANOVA, and variables with skewed distribution was assessed by non-parametric tests. All baseline parameters and treatment data were compared by the univariable Cox proportional hazards model between survivors and deceased. Candidate predictors with P < 0.1 were included in the multivariable Cox regression. The four-category-GGO score was introduced as a fixed variable, and other variables were selected by a forward stepwise (likelihood ratio) procedure based on the P-value. Cumulative survival for different groups was described by Cox regression.

Data analysis was performed with SPSS (version 26.0, USA). P-value < 0.05 was considered significant. GraphPad Prism (Version 6.0, USA) was used for graphing.

## Results

### Cohort description

A total of 200 eligible patients were included for the analysis. Of those, 33.5% of patients died cumulatively within a median follow-up of 15 months from baseline. This retrospective cohort was female predominant (68.5%), with a median age of 52 years (46, 59) and a median disease course of 2 months on admission. The comparison of demographic and clinical features, laboratory results, treatment and outcomes among the four-category-GGO groups were recorded in Table 1. There were no significant differences in gender, disease course, positive anti-Ro52, creatine kinase (CK) and serum ferritin (SF) level among four groups, but age, KL-6 level, and RPILD proportion were increased in groups with higher GGO scores. Mortality in 3, 6, 12 months and all follow-up was increased significantly with GGO scale increased. The mortality during the median follow-up of 15 months were 16.4, 22.2, 48.5, 92.0%, respectively, with an overall mortality of 33.5%. Remission of all patients at 12th month was 35.2%, which was significantly higher in Group A and B.

**Table 1:** Comparison of patients’ baseline characteristics, treatment and prognosis

	Group A (GGO ≤ 1)	Group B (1 < GGO ≤ 2)	Group C (2 < GGO ≤ 3)	Group D (GGO > 3)	All	P-Value
No.(%)	61(30.5)	81(40.5)	33(16.5)	25(12.5)	200(100)	
Demographic features						
Female, n(%)	45(73.8)	53(65.4)	25(75.8)	14(56.0)	137(68.5)	0.289
Age, years, median(IQR) median(IQR)	49(42.5–56.5)	51(45–56)	56(47.5–64.5)	62(52-67.5)	52(46–59)	< 0.001
Disease course, months, median(IQR)	2(1–4)	2(1–4)	2(1–3)	1(1-2.5)	2(1–3)	0.182
RPILD, n( %)	0	37(45.7)	17(51.5)	22(88.0)	76(38.0)	< 0.001
LYMP, × 109/L, median(IQR)	0.74(0.58–1.03)	0.83(0.55–1.12)	0.80(0.61–1.11)	0.60(0.38–1.15)	0.79(0.54–1.05)	0.077
LDH, U/L, median(IQR)	320(258–419)	346(299–437)	348(303–489)	556(358–762)	348(285–447)	< 0.001
CK, U/L, median(IQR) median(IQR)	71(41–131)	72(44.5(151.5)	52(34.5–139)	53(32.5–121)	68(38–135)	0.443
ESR, U/L, median(IQR)	26(13–43)	30(20–48)	43(19–55)	46(29–69)	32(18–50)	0.003
CRP, mg/L, median(IQR)	3.14(1.11–7.55)	6.07(1.51–14.43)	12.10(2.56–22.55)	29.00(11.40-42.39)	6.3(1.56–17.48)	< 0.001
KL-6, U/ml, median(IQR)	628(409–924)	855(649–1209)	1085(827–1666)	2171(1573–2524)	919(611–1316)	< 0.001
SF, ng/ml, median(IQR)	788(285–1547)	959(365–1524)	783(430–1850)	1234(748–2025)	843(399–1664)	0.240
MDA5, U/ml, median(IQR)	165(145–187)	182(164–199)	188(159–213)	178(165–200)	180(159–199)	0.018
Anti-Ro52, n(%)	35((57.4)	53(65.4)	19(59.4)	19(76.0)	126(63.3)	0.387
Treatment						
IVIG, n( %)	19(31.1)	50(61.7)	28(84.8)	19(76.0)	116(58.0)	< 0.001
SMZ Co, n(%)	28(45.9)	56(69.1)	20(60.6)	8(32.0)	112(56.0)	< 0.001
Treatment regimens, n( %)						< 0.001
GC + CTX + CNI	12(19.7)	26(32.1)	11(33.3)	4(16)	53(26.5)	
GC + tofacitinib + CNI	5(8.2)	13(16.0)	3(9.1)	2(8.0)	23(11.5)	
GC + CTX	4(6.6)	16(19.8)	6(18.2)	1(4.0)	27(13.5)	
GC + CNI	22(36.1)	11(13.6)	1(3.0)	2(8.0)	36(18.0)	
Other regimens	18(29.5)	15(18.5)	12(36.4)	16(64.0)	61(30.5)	
Prognosis, n(%)						
Mortality at 3 m	6(10.2)	15(19.0)	15(46.9)	22(88)	58(29.7)	< 0.001
Mortality at 6 m	7(11.9)	16(20.3)	15(46.9)	22(88.0)	60(30.8)	< 0.001
Mortality at 12 m	10(16.9)	17(21.8)	16(51.6)	22(88.0)	65(33.7)	< 0.001
Overall Mortality	10((16.4)	18(22.2)	16(48.5)	23(92.0)	67(33.5)	< 0.001
Remission at 12 months	25(42.4)	35(44.9)	6(19.4)	2(8.0)	68(35.2)	0.001
Severe infection, n(%)	10(16.9)	23(29.1)	7(21.9)	8(32.0)	48(24.6)	0.309

Disease course was defined as the period between the first symptom of DM and hospital admission; RPILD: rapidly progressive interstitial lung disease; LYMP: peripheral blood lymphocyte count; LDH: lactate dehydrogenase; CK: creatine kinase; SF: serum ferritin; ESR: erythrocyte sedimentation, CRP: C-reactive protein; KL-6: Krebs von den Lungen-6; anti-MDA5: anti-melanoma differentiation-associated protein-5; IVIG: intravenous human immunoglobulin; SMZ co: prophylactic use of compound sulfamethoxazole; GC: glucocorticoid; CTX: cyclophosphamide; CNI: calcineurin inhibitor

### HRCT pattern

ILD classifications based on baseline HRCT were shown as Table 2. Nonspecific interstitial pneumonia (NSIP) is the dominant pattern, followed by organizing pneumonia (OP) and NSIP + OP mixture.

**Table 2:** Comparison of patients’ baseline HRCT pattern

Group	GGO ≤ 1	1 < GGO ≤ 2	2 < GGO ≤ 3	GGO > 3	All
n, %	59(30.9)	78(40.8)	33(17.3)	21(11.0)	191(100.0)
ILD type, n(%)					
NSIP, n(%)	46(78.0)	43(55.1)	18(54.5)	9(42.9)	116(60.7)
OP, n(%)	8(13.6)	18(23.1)	9(27.3)	5(23.8)	40(20.9)
NSIP + OP, n(%)	2(3.4)	17(21.8)	6(18.2)	4(19.0)	29(15.2)
NSIP + UIP, n(%)	1(1.7)	0	0	1(4.8)	2(1.0)
UIP + OP, n(%)	0	0	0	2(9.5)	2(1.0)
No ILD, n(%)	2(3.4)	0	0	0	2(1.0)

ILD: interstitial lung disease;NSIP: nonspecific interstitial pneumonia; OP: organizing pneumonia; UIP: usual interstitial pneumonia

### Causes of death

The causes of death were summarized in Table 3. Progression of ILD was the leading cause of death, accounting for 64.2%, followed by infection (25.4%), and mediastinal emphysema (6%). Two patients (3.0%) died of macrophage activation syndrome (MAS) and one patient (1.5%) committed suicide.

**Table 3:** Summary of death causes

Group, n	GGO ≤ 1 (n = 61)	1 < GGO ≤ 2 (n = 81)	2 < GGO ≤ 3 (n = 33)	GGO > 3 (n = 25)	All(n = 200)
Death, n(%)	10(16.4)	18(22.2)	16(48.5)	23(92.0)	67(33.5)
Progression of ILD, n(%)	8(80.0)	8(44.4)	10(62.5)	17(73.9)	43(64.2)
Mediastinal emphysema, n(%)	0	1(5.6)	3(18.8)	0	4(6.0)
Severe infection, n(%)	1(10.0)	8(44.4)	2(12.5)	6(26.1)	17(25.4)
MAS, n(%)	0	1(5.6)	1(6.3)	0	2(3.0)
Other causes, n(%)	1(10.0)	0	0	0	1(1.5)
Total death, n(%)	10	18	16	23	67

### Univariable analysis and determination of cut-off values

Univariate Cox regression showed that age (16–55, > 55y), disease course (1–2, > 2 months), RPILD, KL-6 (0-1000, > 1000 U/ml), LMYP (0-0.6, > 0.6 × 10^9^/L), LDH (0-460, > 460 U/L), SF (0-600, > 600 ng/ml), ESR (0–45, > 45 mm/h), CRP (0–5, > 5 mg/L), positive anti-Ro52, four-category-GGO score, prophylactic use of SMZ Co and five-category-treatment regimens were associated with death (Table 4). Creatine kinase (0–70, > 79U/L) and anti-MDA5 (0-180, > 180U/ml) were not associated with death.

**Table 4:** Initial parameters associated with death using Cox regression model

	Univariate analysis			Multivariate analysis		
	HR	95%CI	p value	HR	95%CI	P value
Age > 55 years	3.064	1.870–5.020	< 0.001	-	-	-
Disease course > 2 months	0.521	0.300-0.904	0.020	-	-	-
RPILD	2.556	1.569–4.164	< 0.001	-	-	-
KL-6 > 1000 U/ml	2.126	1.298–3.480	0.003	-	-	-
LYMP > 0.6 × 10^9^/L	0.481	0.297–0.779	0.003	-	-	-
LDH > 460 U/L	3.499	2.150–5.695	< 0.001	-	-	-
CK > 70 U/L	1.099	0.681–1.775	0.699	-	-	-
SF > 600 ng/ml	3.206	1.677–6.128	< 0.001	2.277	1.136–4.564	0.020
ESR > 45 mm/h	2.215	1.367–3.587	0.001	-	-	-
CRP > 5 mg/L	4.000	2.177–7.348	< 0.001	2.512	1.219–5.177	0.013
Anti-Ro52 positive	2.526	1.376–4.637	0.003	2.295	1.228–4.289	0.009
Anti-MDA5 > 180U/ml	0.990	0.613–1.599	0.969	-	-	-
Groups (compared with A)			< 0.001	-	-	-
Group B	1.383	0.639–2.997	0.411	1.408	0.626–3.166	0.408
Group C	3.484	1.577–7.698	0.002	3.433	1.464–8.047	0.005
Group D	8.315	3.882–17.811	< 0.001	4.376	1.897–10.097	0.001
IVIG	1.378	0.835–2.272	0.210	-	-	-
SMZ Co	0.318	0.189–0.533	< 0.001	0.315	0.178–0.556	< 0.001
Treatment regimens			0.029	-	-	-
Compared with GC + CTX + CNIs + CGC + CTX + CNI						
GC + tofacitinib + CNI	0.865	0.308–2.425	0.782			
GC + CTX	1.569	0.687–3.582	0.285			
GC + CNI	0.887	0.368–2.142	0.790			
Other regimens	2.240	1.171–4.286	0.015			

RPILD: rapidly progressive interstitial lung disease; KL-6: Krebs von den Lungen-6; LYMP: peripheral blood lymphocyte count; LDH: lactate dehydrogenase; CK: creatine kinase; SF: serum ferritin; ESR: erythrocyte sedimentation, CRP: C-reactive protein; anti-MDA5: anti-melanoma differentiation-associated protein-5; Group A, GGO ≤ 1; Group B, 1 < GGO ≤ 2; Group C, 2 < GGO ≤ 3; Group D, GGO > 3; IVIG: intravenous human immunoglobulin; SMZ co: prophylactic use of compound sulfamethoxazole; GC: glucocorticoid; CTX: cyclophosphamide; CNI: calcineurin inhibitor

Finally, 13 candidate predictors were chosen: age > 55y, disease course > 2 months, RPILD, KL-6 > 1000 U/ml, LMYP > 0.6 × 10^9^/L, LDH > 460 U/L, SF > 600 ng/ml, ESR > 45 mm/h, CRP > 5 mg/L, anti-Ro52, four-category-GGO score, prophylactic use of SMZ Co and five-category-treatment regimens, and they were included in multivariate Cox regression (Table 4).

### Multivariable analysis and determination of final prognostic predictor

**Fig. 1:**
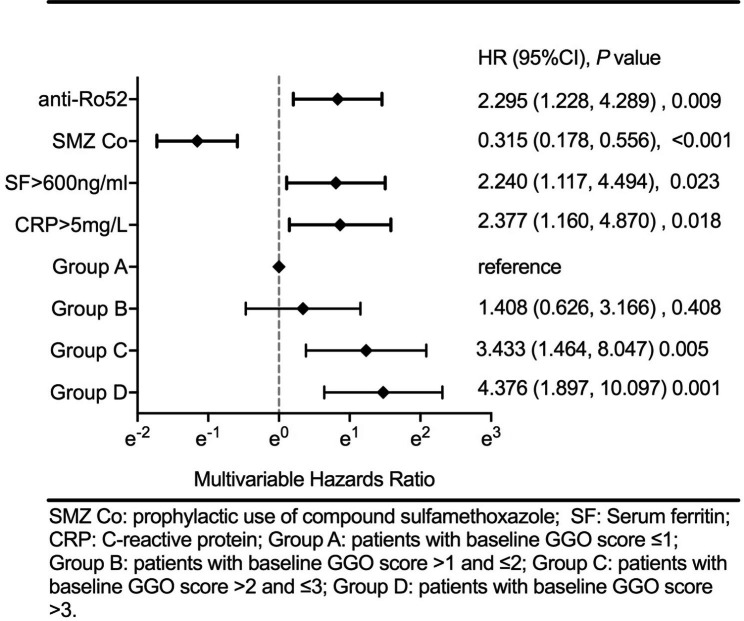
Independent risk factors of death for patients with anti-MDA5-DM.

The 13 variables screened out with P < 0.1 in univariate analysis were included in multivariate analysis, among which, the four-category GGO was introduced as a fixed variable, and other variables were selected by a forward stepwise (likelihood ratio) procedure based on the P-value. In the final multivariable Cox regression models, high level GGO score, CRP > 5 mg/L, positive anti-Ro52 and SF > 600 ng/ml were determined as independent risk factors for mortality of anti-MDA5-DM, and prophylactic use of SMZ Co were determined as an independent protective factor (Table 4). Compared with Group A, the mortality of Group B was not significantly increased. In contrast, the mortality of Group C increased 2.433 times (exp(B) = 3.433, p = 0.005, [1.464, 8.047]), and the mortality of Group D increased 3.376 times (exp(B) = 4.376, p = 0.001, [1.897, 10.097]) (Fig. 1). The mortality of patients with CRP > 5 mg/L increased 1.377 times (exp(B) = 2.377, p = 0.018, [1.160, 4.870]). The mortality of patients with SF > 600 ng/ml increased 1.240 times (exp(B) = 2.240, p = 0.023, [1.117, 4.494]). The mortality of patients with positive anti-Ro52 increased 1.295 times (exp(B) = 2.295, p = 0.009, [1.228, 4.289]). The mortality of patients who took prophylactic use of SMZ Co were significantly decreased (exp(B) = 0.315, p < 0.001, [0.178, 0.556]).

**Fig. 2:**
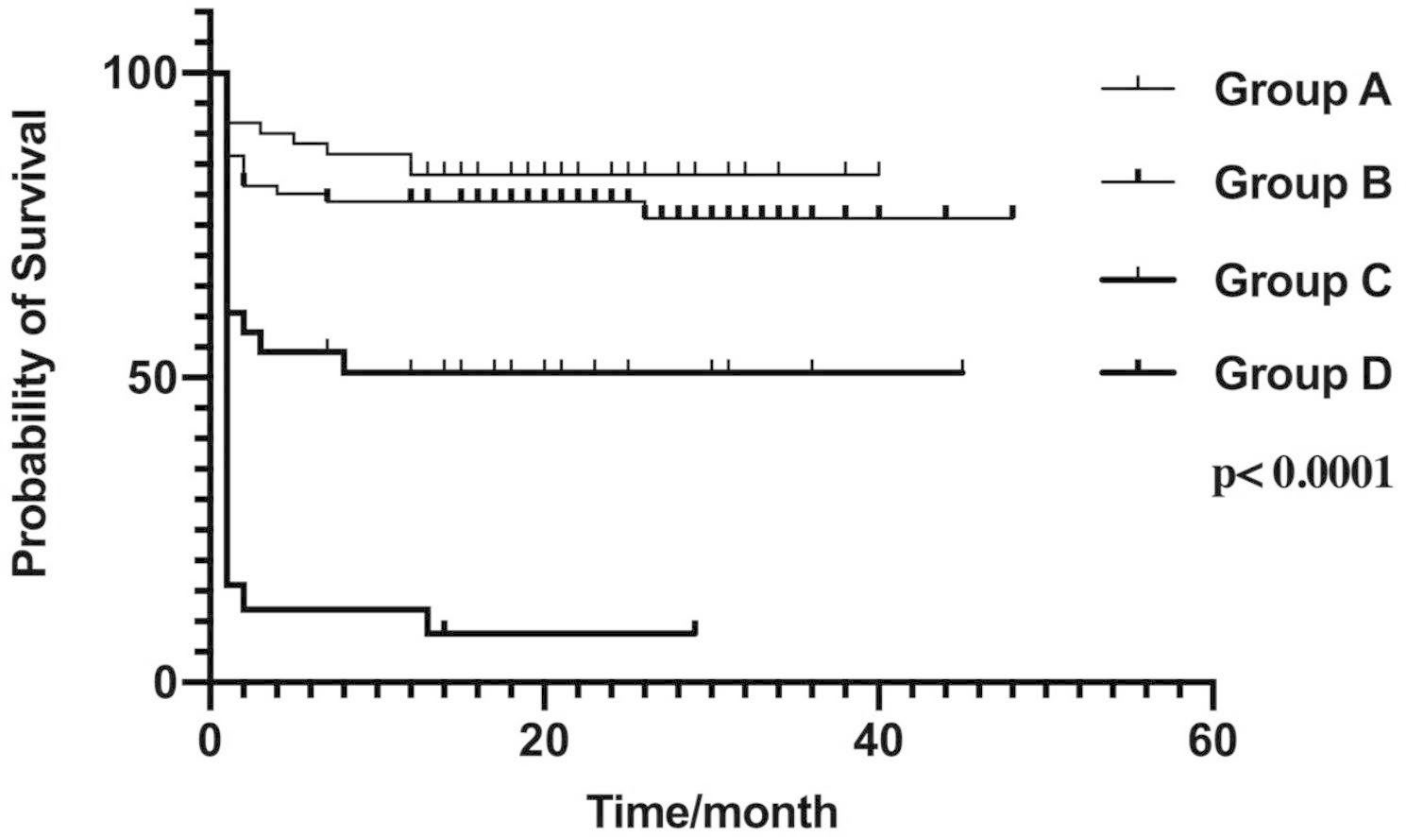
Comparision of cumulative survival rate between different groups

The Cox survival curves were shown in Fig. 2. The cumulative survival rate of Group A was higher, followed by Group B, Group C, and then Group D.

## Discussion

This study was based on a large single-centre cohort and several prognostic factors of anti-MDA5-DM were identified. GGO-based categorical parameter (GGO ≤ 1, 1 < GGO ≤ 2, 2 < GGO ≤ 3, GGO > 3) was a reliable predictor of all-cause mortality. Previous studies have also explored the association between HRCT scores and prognosis in anti MDA5-DM, but the number of cases was relatively small [7, 12, 16, 21].

Accumulating data suggest that higher serum ferritin levels are associated with poor prognosis in anti-MDA5-DM [5, 9, 15, 16]. Our study made similar observations although the cut-off value was different. In other studies, the cut-off values set the critical value as 450, 1000 or 1500 ng/ml [7, 16, 21], while the critical value we obtained through the ROC curve is 591.2 ng/ml.

The titer of anti-MDA5 antibody is useful for the evaluation of the treatment response in anti-MDA5-DM, and resistance to treatment, and sustained high levels of anti-MDA5 antibody were present in patients who died [5]. It has been shown that the titer of anti-MDA5 antibody may be used to predict outcomes of RPILD as well as to monitor disease activity [26]. Another study demonstrates that higher titer of anti-MDA5 antibody is an independent risk factor of RPILD [10]. Inconsistent with their findings, we did not find an association of anti-MDA5 titer with either RPILD or mortality. However, we did notice that rapidly decreased level of anti-MDA5 antibody was often accompanied by remission, and re-elevation of anti-MDA5 antibody was often accompanied by relapse. Therefore, we posit that the titer of anti-MDA5 antibody may be related to disease activity.

A previous report shows that high-level KL-6 is associated with poor prognosis in anti-MDA5-DM patients [7]. Similar results were obtained from our study after univariate analysis, although the difference was not significant in multivariate analysis. Bivariate correlation analysis revealed that KL-6 and GGO scores were significantly correlated. Therefore, KL-6 was not considered an independent risk factor of mortality.

Previous studies have demonstrated that positive anti-Ro-52 and higher CRP were poor prognostic factors for anti-MDA5-DM patients [7, 10, 13], which is consistent with our results.

Successful treatment of patients with anti-MDA5-DM, particularly, those with RPILD, poses a tremendous challenge to rheumatology society. A previous study shows that a combination of high-dose GC and CNIs with or without CYC is the first choice [17]. Previous studies indicate that a triple combination of high-dose GC with CYC and CNI significantly improves the prognosis of patients [9]. Adding tofacitinib to basic treatments may bring refractory anti-MDA5-DM under control [21]. Accordantly, Chen, et al. also demonstrate that tofacitinib benefits patients with anti-MDA5-DM [20]. Our study compared GC + CTX + CNI, GC + tofacitinib + CNI, GC + CTX, GC + CNI and other regimens, and did not detect statistical difference among five groups in mortality. It should be pointed out that the baseline GGO score of GC + CNI group was significantly lower than the other groups. It is reasonable to draw the conclusion that GC + CNI is less effective than other regimens in anti-MDA5-DM.

As pneumocystis jirovecii infection (PJP) is common in anti-MDA5-DM, effective prevention of PJP is crucial for reducing the mortality [27]. This study revealed prophylactic use of SMZ Co may significantly decrease risk of mortality in anti-MDA5-DM, thus, we strongly recommend prophylactic use of SMZ Co.

Our study is a real-world study that included a large number of anti-MDA5-DM patients, which may shed light on the risk stratification anti-MDA5-DM. However, this study has its limitations. Firstly, it is a single centered retrospective study, multi-centered investigation may improve our current findings. Secondly, it was not a randomized controlled clinical study, and further studies are needed to consolidate our observations.

In conclusion, our study investigated the risk factors for all-cause death, and explored the value of four-category-GGO score as a prognostic basis in anti-MDA5-DM. GGO-based categorical parameter (GGO ≤ 1, 1 < GGO ≤ 2, 2 < GGO ≤ 3, GGO > 3) was a reliable predictor of all-cause mortality.

## Data Availability

Data sets generated during the current study are available from the corresponding author on reasonable request, but restrictions apply to the availability of these data, which were used under license for the current study, and so are not publicly available.

## Abbreviations

ALB: Albumin
anti-MDA5: Anti-melanoma differentiation-associated protein-5 antibody
CK: Creatine kinase
CNI: Calcineurin inhibitors
CRP: C-reactive protein
CYC: Cyclophosphamide
DM: Dermatomyositis
ESR: Erythrocyte sedimentation rate
GC: Glucocorticoids
GGO: Ground-glass opacity
HR: Hazard ratio
HRCT: High-resolution CT
ILD: Interstitial lung disease
IQR: Interquartile range
IVIG: Intravenous immunoglobulin
KL-6: Krebs von den Lungen-6
LDH: Lactate dehydrogenase
RPILD: Rapidly progressive interstitial lung disease
SD: Standard deviation
SF: Serum ferritin
SMZ Co: Compound sulfamethoxazole
